# Expert consensus on the prevention, diagnosis and treatment of cold injury in China, 2020

**DOI:** 10.1186/s40779-020-00295-z

**Published:** 2021-01-21

**Authors:** Hong-Xu Jin, Yue Teng, Jing Dai, Xiao-Dong Zhao, Members of the Emergency Medicine Committee of the People’s Liberation Army, Yang Cao, Cong Chen, Wei Chen, Chen-fang Fan, Jun Fei, Le-Wen Gan, Ying-Fei Guo, Jian He, Zhi-Gang Huang, Bin-Long Ji, Nan Li, Wei-Qin Li, Hong-Sheng Liu, Ming-Hua Liu, Shuang-Qing Liu, Ya-Hua Liu, Yu-Peng Liu, Dong Lv, Rui-Heng Ma, Shi-Nan Nie, Xian-Feng Pan, Yi Shan, Kai-Jin Shen, Zhong Shi, Hai-Jing Song, Zu-Jun Song, Qin Su, Li-Dong Sun, Shao-Hui Tang, You-Qing Tang, Zhong-Zhi Tang, Xiao-Xi Tian, Bo-Liang Wang, Qi Wang, Qian Wang, Man Wang, Song-Tao Wang, Yu-Hong Wang, Qiang Xiang, Gui-Sen Xu, Shuo-Gui Xu, Wen-Xin Yang, Yong-Hong Yao, Ming Yin, Wen Yin, Zong-Wei Yin, Fu-Bin Zhang, Jian-Bo Zhang, Wei Zhang, Xi-Gang Zhang, Yu Zhang, Fei-Hu Zhou, Hong Zhou, Ren-Jie Zhou, Rong-Bin Zhou, Yue-Su Zhou, Hai-Yan Zhu

**Affiliations:** 1 Emergency Medicine Department, General Hospital of the Northern Theater Command, Shenyang, 110016 China; 2 Department of Emergency Medicine, the Fourth Medical Center, Chinese PLA General Hospital, Beijing, 100048 China

**Keywords:** Cold injury, FCI, NFCI, Treatment, Expert consensus

## Abstract

Cold injury refers to local or systemic injury caused by a rapid, massive loss of body heat in a cold environment. The incidence of cold injury is high. However, the current situation regarding the diagnosis and treatment of cold injury in our country is not ideal. To standardize and improve the level of clinical diagnosis and treatment of cold injury in China, it is necessary to make a consensus that is practical and adapted to the conditions in China. We used the latest population-level epidemiological and clinical research data, combined with relevant literature from China and foreign countries. The consensus was developed by a joint committee of multidisciplinary experts. This expert consensus addresses the epidemiology, diagnosis, on-site emergency procedures, in-hospital treatment, and prevention of cold injury.

## Background

Cold injury refers to local or systemic injury caused by a rapid, massive loss of body heat in the cold environment. Cold injury is a common disease in cold regions and plateau cold regions that can occur in daily life and in production and military activities. The incidence of cold injury is high, but the current situation of diagnosis and treatment of cold injury in our country is not ideal. The implementation of standardized treatment for cold injury is in line with China’s national conditions and social development needs. It is of great significance that the level of clinical diagnosis and treatment of cold injury be standardized and improved to ensure the health of our people, especially those in cold areas.

Currently, the literature related to the diagnosis and treatment of cold injury mostly comes from military activities, and the study types are mainly retrospective analyses, literature reviews, and expert opinions, which lack systematic clinical diagnosis and treatment guidelines. Therefore, to actively address cold injuries in China, a joint committee composed of multidisciplinary experts jointly formulated the *Consensus of experts on the prevention, diagnosis and treatment of cold injury in China in 2020* based on the latest population-level epidemiological, clinical research data and relevant literature, aiming to guide the treatment of cold-related injury in China. The recommendations in the consensus were graded by the group of experts based on the quality of the literature, data and the balance between the benefits, risks and costs of each proposal. The grading is based on the American College of Chest Physicians (ACCP) formulation of recommended levels for clinical guidelines (Table 1).

**Table 1:** Grading of recommendations

Grade of recommendation	Clarity of risk/benefit	Quality of supporting evidence	Implication
1A: Strong recommendation, high-quality evidence	Benefits clearly outweigh the risks and burdens, or vice versa	RCTs without important limitations or overwhelming evidence from observational studies	Strong recommendation, applies to most patients in most circumstances without reservation
1B: Strong recommendation, moderate-quality evidence	Benefits clearly outweigh the risks and burdens, or vice versa	RCTs with important limitations (inconsistent results, methodological flaws, indirect analyses, or imprecise conclusions) or exceptionally strong evidence from observational studies	Strong recommendation, applies to most patients in most circumstances without reservation
1C: Strong recommendation, low-quality or very low-quality evidence	Benefits clearly outweigh the risks and burdens, or vice versa	Observational studies or case series	Strong recommendation but subject to change when higher quality evidence becomes available
2A: Weak recommendation, high-quality evidence	Benefits closely balanced with the risks and burden	RCTs without important limitations or overwhelming evidence from observational studies	Weak recommendation, best action may differ depending on the patient, treatment circumstances, or social values
2B: Weak recommendation, moderate-quality evidence	Benefits closely balanced with the risks and burden	RCTs with important limitations (inconsistent results, methodological flaws, indirect or imprecise) or exceptionally strong evidence from observational studies	Weak recommendation, best action may differ depending on the patient, treatment circumstances, or social values
2C: Weak recommendation, low-quality or very low-quality evidence	Uncertainty in the estimates of the benefits, risks, and burden; benefits, risk, and burden may be closely balanced	Observational studies or case series	Very weak recommendation; alternative treatments may be equally reasonable and merit consideration

## Classification and grading

### Classification and mechanism

Cold injury can be divided into systemic cold injury and local cold injury.

Systemic cold injury, also known as low temperature or accidental hypothermia, refers to the continuous loss of heat after cold exposure, and the core temperature gradually decreases to less than 35.0 °C.

According to its nature, local cold injury can be divided into two types: freezing cold injury (FCI) and non-freezing cold injury (NFCI). It is generally believed that FCI occurs when the tissue is exposed to a freezing point of approximately 0.55 °C; the ambient temperature is usually below 0 °C, and there is tissue freeze-thaw injury. If tissue is exposed to a cold and humid environment for a long time (hours to days) that is not enough to cause freezing, NFCI easily occurs. At this time, the ambient temperature is usually between 0 °C and 10 °C [1]. It has also been reported that cold injury may occur at 15 °C or higher [2]. There is no tissue freeze-thaw injury in NFCI.

The injury mechanisms of the different kinds of cold injury are different. Systemic cold injury, for example, is mainly related to a decrease in the enzyme activity that maintains normal human tissue metabolism and is complicated by multiple organ dysfunction, affecting the brain, lung, liver, kidney, and so on. It is often characterized by hypothermia, which is accompanied by a lack of alertness and even loss of consciousness in clinical practice [3].

NFCI is often caused by ischemia-reperfusion injury. Ochrodermia and paresthesia can be manifested in the early stage of the disease. There may be hyperemia and edema, accompanied by severe burning-like pain, after rewarming. NFCI is characterized by continuous hypersensitivity to cold sensation after the removal of stimulation. FCI is mainly caused by vascular endothelial injury resulting from freeze-thaw injury, which involves cell dehydration and mechanical injury, accompanied by local microcirculatory disorders. FCI usually occurs in the hands, feet, ears, nose and other parts. Its clinical manifestations are similar to those of NFCI. However, after rewarming, the sensation often weakens or even disappears, and there may be bloody or serous blister formation. The lesions often have ulceration after scabbing, even secondary infection, gangrene and other manifestations.

**Recommendation 1: Cold injury can be divided into systemic cold injury and local cold injury, and local cold injury can be divided into FCI and NFCI. (Grade 1A).**

### Local cold injury

#### *FCI*

Generally, according to the grading scheme of thermal burns, local cold injury can be divided into four stages. This grading scheme is based on limb performance with frostbite and further changes after rewarming [4] (Additional file 1: Appendix reference form 1). The classification is suitable for FCI. At the same time, the tissue in the state of frostbite is hard, pale and sensationless. It is difficult to evaluate the degree of cold injury on-site or before rewarming of the limb. Therefore, in 2001, the Wilderness Medical Society (WMS) proposed using a backup classification [5] that is more suitable for on-site treatment (Table 2).

**Table 2:** Simple classification of cold injuries from WMS

Level	Content
Superficial	No or minimal anticipated tissue loss, corresponding to 1st- and 2nd-degree injury
Deep	Deeper injury and anticipated tissue loss, corresponding to 3rd- and 4th- degree injury

*WMS* Wilderness Medical Society

#### *NFCI*

NFCI is mainly concentrated in the limbs, especially the feet, so it was also initially known as *trench foot*, which may be related to long exposures to wet and cold environments [6]. Worldwide (especially in the military), the Webster classification (divided into four grades: lightest/light/moderate/severe, according to the clinical manifestations of the foot 2–3 days after injury) and the Ungley classification (divided into four grades: A\B\C\D, according to the distribution of sensory loss area 7 days after injury) are often used for grading [7]. As the outcomes suggested by the two methods are almost the same, the two classification schemes are often combined in the clinic (Table 3).

**Table 3:** Ungley and Webster grading schemes combined

Stage	Clinical manifestation	Ending
Lightest/A	Hyperemia and mild sensory abnormalities lasted for 2–3 days, and there were no signs and sensory abnormalities 7 days after the injury.	Generally, the walking ability can be restored quickly; the working ability can be restored in 1–2 weeks. The sensitivity to cold is occasionally increased.
Light /B	Edema, hyperemia and disappearance of sensation are still found 2–3 days after injury; 7 days after the injury, there is still abnormal sensation on the foot bottom and at the toe tip, which lasts for 4–9 weeks. No blisters or skin loss.	If the pain is not aggravated, the patient can walk; it will take 3 to 4 months to restore the working ability and hyperhidrosis. Increased cold sensitivity will remain in some patients.
Medium/C	There are still edema, hyperemia and blisters 2–3 days after injury. On the 7th day after injury, the tactile sense of the dorsum, sole and toe of the foot is not recovered, vibration and position sense are weakened, and the muscle of foot is atrophied. Edema will last 2–3 weeks, pain and hyperemia can last 2–3 months, and there is no loss of deep tissue.	In the short term, most patients need to walk with the help of equipment or others; it takes more than 6 months to 1 year to restore their working ability, and their ability to do fine motor skills is lessened or even lost. Most patients will still have hyperhidrosis and cold-sensitivity, and some will have permanent disabilities.
Severe/D	Severe edema and exudation occur 2–3 days after injury. Seven days after injury, there is still a total loss of foot sensation, muscle paralysis and atrophy, and the injury often reaches above the foot. Tissue edema lasts for 3–7 weeks, and the hyperemia and pain can last for 4 months. Tissue loss may be caused by spontaneous amputation of limbs, and gangrene is more common.	Patients needed to walk with the help of equipment or others; they are often left with permanent disabilities and rarely continue to work.

### Systemic cold injury

Systemic cold injury is mainly related to the overall pathophysiological changes caused by a decrease in core body temperature and is often graded according to the core body temperature (Appendix reference form 2). In general, the systemic circulation blood temperature is the most accurate, but it involves an invasive monitoring method. Among the following common noninvasive methods, esophageal monitoring reflects the central body temperature most accurately because it is located inside the body cavity and close to the heart; however, it should be monitored 1/3 of the way down the esophagus, and inhaling warm gas may interfere with its accuracy. Another common method of temperature measurement is rectal temperature measurement, which is taken at a depth of at least 15 cm, but rewarming will delay the reflection of core body temperature. Tympanic membrane temperature measurement can accurately reflect intracranial temperature. However, it is necessary to ensure that the ear canal is clean and isolated from the outside.

Under the condition of on-site emergency treatments, it is difficult to obtain a core body temperature quickly. Therefore, the *Swiss Classification* has emerged, and in it, systemic cold injury is divided into 4 stages according to symptoms and signs, such as shivering, changes in consciousness and vital signs [8] (Table 4).

**Table 4:** Staging of accidental hypothermia in Switzerland

Stage	Clinical presentation	Typical core temperature
HT I	Conscious, shivering	35 °C to 32 °C
HT II	Impaired consciousness, not shivering	< 32 °C to 28 °C
HT III	Unconscious, not shivering, vital signs present	< 28 °C to 24 °C
HT IV	No vital signs	< 24 °C

*HT* Hypothermia.

**Recommendation 2: Systemic cold injury is usually classified according to core body temperature, and local cold injury is classified according to the depth of tissue injury. Each type of cold injury has its own classification. (Grade 1B).**

## Epidemiology

Generally, the occurrence of cold injury is closely related to the ambient temperature during work and life. Cases related to cold injury are rarely reported in middle and low latitudes; in areas with a wide range in latitude between the north and south, such as the United States, the incidence of cold injury in a military survey is approximately 0.03% [9]. It is worth noting that in another study, the incidence of cold injury among 1080 Marines during missions in Norway was as high as 5% [10]. In some high-latitude countries, such as Finland, the incidence of cold injury among recruits can rise to 0.18% [2]. At higher latitudes, such as Antarctica, the incidence among researchers can be as high as 6.56% [1].

Additionally, the occurrence of frostbite is also related to occupation. In the past, it was generally believed that soldiers, fishermen and cold store workers (such as meat processors) were at high risk of cold injury. For example, a study in Finland showed that the lifetime incidence rate in soldiers was as high as 44%, and the annual incidence rate was approximately 2.2% [11]. A study on cold injury in soldiers in northeast China also suggested that the overall incidence was approximately 40.7% [12]. Recent studies have also found that the incidence in mountaineering (high mountain or snow-capped mountain) enthusiasts has also reached 36.6% [13]. The lifetime incidence rate among herders in cold areas (such as reindeer herders) is approximately 65% [13]. In addition, it should be noted that some people with poor awareness regarding cold protection, such as students, farmers, and homeless people, also have a high incidence of cold injury [14].

In wartime, the incidence of cold injury often shows an explosive increase. For example, during World War II, the number of US troops with frostbite was as high as 91,000, while during the Korean War, 6300 people were frostbitten (49% of them had frostbite limb necrosis, and 6% had distal tissue loss) [15]. On the other hand, in the Soviet-Finnish War of 1939 and 1940, the number of Soviet frostbite cases reached 17,867, accounting for 6.7% of all casualties; in Finland, there are approximately 7900 cases of cold injury, accounting for 12% of the casualties [16]. It needs to be clear that because of updates in medical concepts, technological progress and changes in the form of war, these data are not representative of the current actual situation. For example, during the winter military activities of the US military during the war in Afghanistan, the number of cold injuries was only 19, and the incidence rate was approximately 0.1%, which was significantly lower than that during World War II [15].

The effect of sex on cold injury remains to be studied. A retrospective analysis in the United States showed that the male-to-female ratio was 10:1 [17]. However, the data mainly come from army and winter athletes (such as skiers), where there is a significant difference in the male-to-female ratio. Another study based on hospitalization data showed that men of all ages had a higher incidence than women, and the total number of men was significantly higher than that of women (it is worth mentioning that the incidence rate of the civilian population is approximately 2.5/100,000) (Appendix reference form 3) [18]. This conclusion is consistent with the results of some retrospective case analyses in China: in the study of Niu et al. [19], 277 of 397 patients with cold injury were male, and their ages ranged from 3 to 78 years. However, some researchers have pointed out that there is no significant correlation between sex and the occurrence of cold injury. As reported by DeGroot et al. [20], taking into account all races, the number of men and women with cold injuries per 100,000 people is similar (13.9:13.3).

**Recommendation 3: The occurrence of cold injury is closely related to the ambient temperature where patients live and work, and the incidence of cold injury is high in military activities. (Grade 1C).**

## Prevention

Generally, cold injury is preventable, but environmental, psychological and health problems must be addressed at the same time. Cold injury occurs when the heat loss of tissue exceeds the ability of tissue perfusion to prevent freezing. To avoid frostbite, adequate perfusion must be ensured, and heat loss must be minimized.

### Maintain adequate tissue perfusion

Tissue perfusion should be maintained as follows:

1. Maintain an adequate core temperature, such as by adding an appropriate amount of clothing;
2. Perform appropriate activities. In a small study, when the toes were immersed in cold water after exercise, compared with 28% of the control group, 58% of the subjects observed peripheral vascular dilatation in the toes. This may be related to the increase in core body temperature after activity [21]. Of course, excessive exercise will cause fatigue of the body, and the secretion of sweat will also destroy the dry environment on the body surface, resulting in substantial heat loss and a drop in core temperature. Given this, the occurrence of cold injury can be prevented by raising the core body temperature through appropriate activities;
3. Minimize the effects of underlying diseases or drugs that may reduce peripheral tissue perfusion (such as cardiac insufficiency or the use of epinephrine, ephedrine and other drugs) and avoid working in cold areas as much as possible;
4. Cover all skin (including head and face) with clothing to avoid vasoconstriction stimulated by the cold environment;
5. Minimize restrictions on blood flow, such as by removing tight clothing or shoes or by moving the limbs;
6. Ensure adequate water and energy/nutrition supply and intake.

### Reduce the exposure of tissue to a cold environment

Measures should be taken to minimize tissue exposure. These measures include the following:

1. Avoid environmental conditions with a risk of causing cold injury, especially those below − 15 °C (even if the wind speed is very low) [22];
2. Avoid dampness, cold and wind on the skin;
3. Avoid sweating or damp limbs, especially feet;
4. Ensure that appropriate responses are made to changing environmental conditions or scenes (for example, clothes can be appropriately removed when participating in physical activities and added after the end of the activities to avoid increased fatigue or sweating caused by bulky clothing);
5. Use heating equipment;
6. Shorten the cold exposure time as much as possible.

It must be noted that emollients do not prevent cold injury and may even increase the risk of cold injury [23]. Limb sensory abnormalities should be addressed in a timely manner because we cannot assess the maximum acceptable time of numbness or other abnormal sensations at the end of the limb before suffering a cold injury. Limbs at risk of frostbite (such as those with numbness, poor flexibility, pale skin) should be rewarmed by using their own or a partner’s armpit or abdominal temperature.

### Appropriate cold acclimatization

Cold acclimatization can be carried out if necessary. For a long time (usually 4–6 weeks), within the limits of physiological tolerance, repeated cold stimulation can induce a gradual weakening of the cold stress response and an obvious enhancement of cold tolerance and antifreeze ability. However, some studies have pointed out that prolonged cold acclimatization weakens the sense of touch, temperature and vibration of the limbs and increases the severity and universality of foot symptoms and pain [24]. Therefore, cold acclimatization should be carried out as appropriate.

**Recommendation 4: The prevention of cold injury includes maintaining tissue perfusion, reducing tissue exposure to cold environments, and ensuring proper cold acclimatization. (Grade 1B).**

## Diagnosis and treatment

It is not very difficult to diagnose systemic cold injury according to the patient’s core body temperature or state of consciousness. However, in the initial stage, it may be difficult to determine the degree of injury or distinguish FCI from NFCI. Deep tissue damage often takes weeks to fully manifest. Moreover, due to individual pathogenic factors, two types of local cold injury may coexist in the same patient or even the same limb, which also increases the difficulty of diagnosis.

Under on-site conditions, the diagnosis of local cold injury can be determined simply by rewarming via the armpits, abdomen or other warm areas for approximately 30 min. If the temperature of the affected site recovers, the symptoms are completely relieved, and there is no change in skin color or sensory abnormality, the patient can be considered to have no obvious symptoms of local cold injury and need no treatment other than close observation. Nevertheless, if the symptoms do not recover obviously or frostbite occurs again, local cold injury should be noted and treated accordingly [25]. It should be mentioned that if there is no definite evidence of tissue freezing injury, the patient should be treated for NFCI.

When all types of cold injury occur in the same patient, priority should be given to the treatment of systemic cold injury, and local cold injury could be treated at the same time (or later). The treatment sequence for FCI is administered prior to that for NFCI.

**Recommendation 5: The type of cold injury should be defined during the treatment. The sequence of treatment should be systemic cold injury—FCI (at the same time or after)—NFCI. (Grade 1C).**

### On-site emergency treatments

#### Systemic cold injury

During first aid on-site, it is necessary to get the patient out of the cold environment as soon as possible and wrap the body with blankets or clothing to keep warm. Because the vital signs of these patients are often weak, the vital signs should be checked every minute if there is no vital monitoring equipment, especially the pulse. If the pulse cannot be measured, cardiopulmonary resuscitation (CPR) should be started immediately, and the patient should be sent to the hospital for further treatment. If possible, rewarming can be started synchronously, but it should not affect the progress of CPR or transfer work. In addition, if a patient has a cold injury resulting from drowning or an avalanche, tracheal intubation should be actively performed early. Inhaling warm gas is an optional method for core rewarming.

**Fig. 1:**
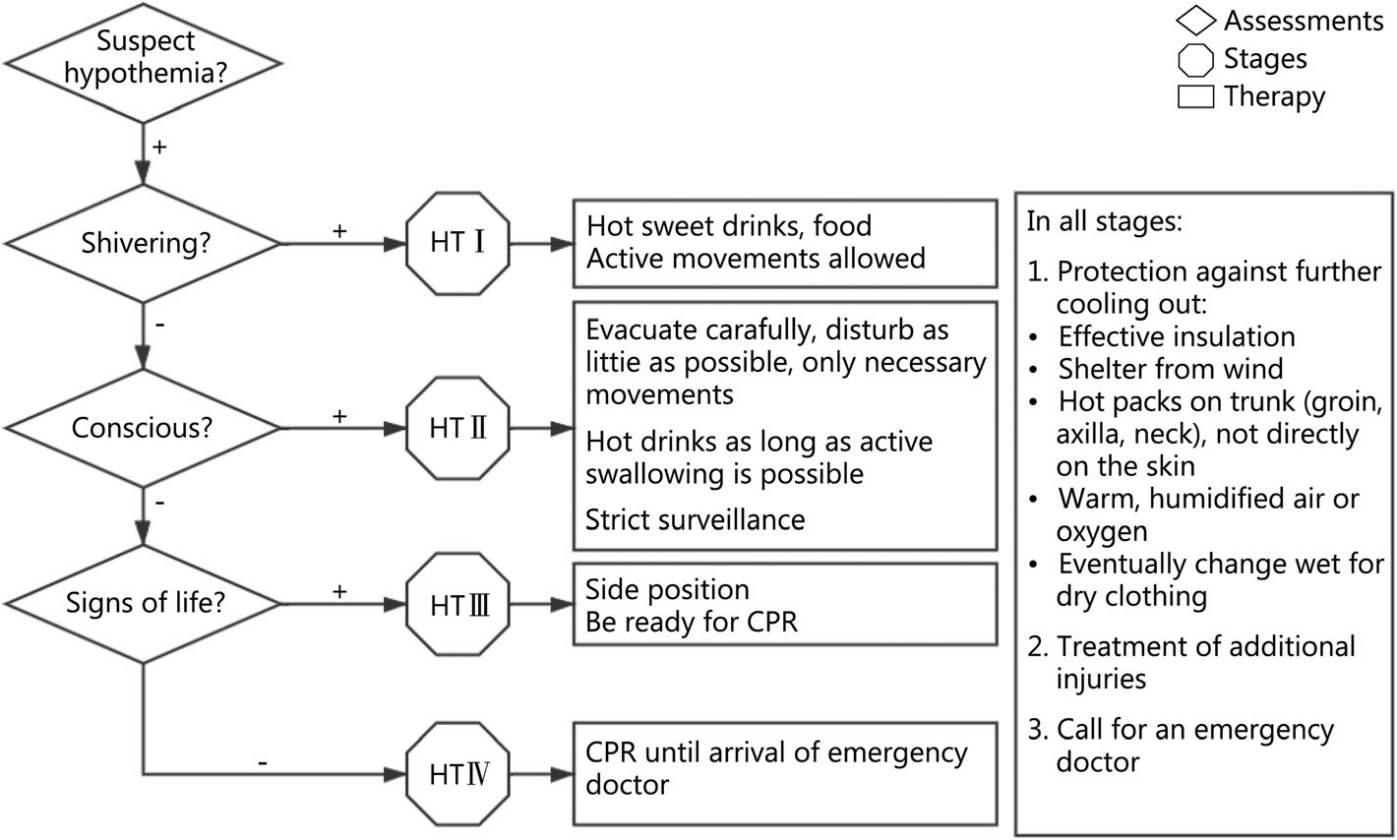
On-site emergency treatments for systemic cold injury. HT: Hypothermia; CPR: Cardiopulmonary resuscitation

Patients with different grades of systemic cold injury are given treatment according to the Swiss grading system for on-site emergency treatment for systemic cold injury [26] (Fig. 1).

In addition, due to the contraction of peripheral blood vessels caused by hypothermia, insufficient blood volume circulation and a decrease in temperature-induced release of antidiuretic hormone, most patients will have different degrees of hypovolemia. Therefore, fluid resuscitation or volume therapy is common and essential. Infusion of physiological saline alone may aggravate acidosis, so it is emphasized that both sugar and salt should be taken into account, and disorders of water and electrolyte metabolism and changes in acid-base balance should be avoided at the same time. It is suggested that the infusion of liquid should be heated (generally to less than 42 °C), and rewarming can be achieved at the same time.

Before transfer, the patient’s condition, vital signs, core body temperature and serum potassium level should be assessed to determine the appropriate medical unit level and qualifications. At the same time, hypothermia affects the basic metabolic rate of patients and improves the tolerance of the body in regard to ischemia and hypoxia, so it is not appropriate to terminate CPR prematurely. In fact, among the reported cases of cardiac arrest caused by systemic cold injury, the longest time for successful treatment with CPR was 190 min (relying solely on external rewarming), and it can even reach 390 min when using a rewarming blanket and intrapulmonary perfusion [27].

**Recommendation 6: On-site emergency treatments of systemic cold injury should include evaluation grading and close monitoring of vital signs. CPR should be performed immediately if necessary, and CPR should not be terminated prematurely. (Grade 1C).**

#### Local cold injury

**Fig. 2:**
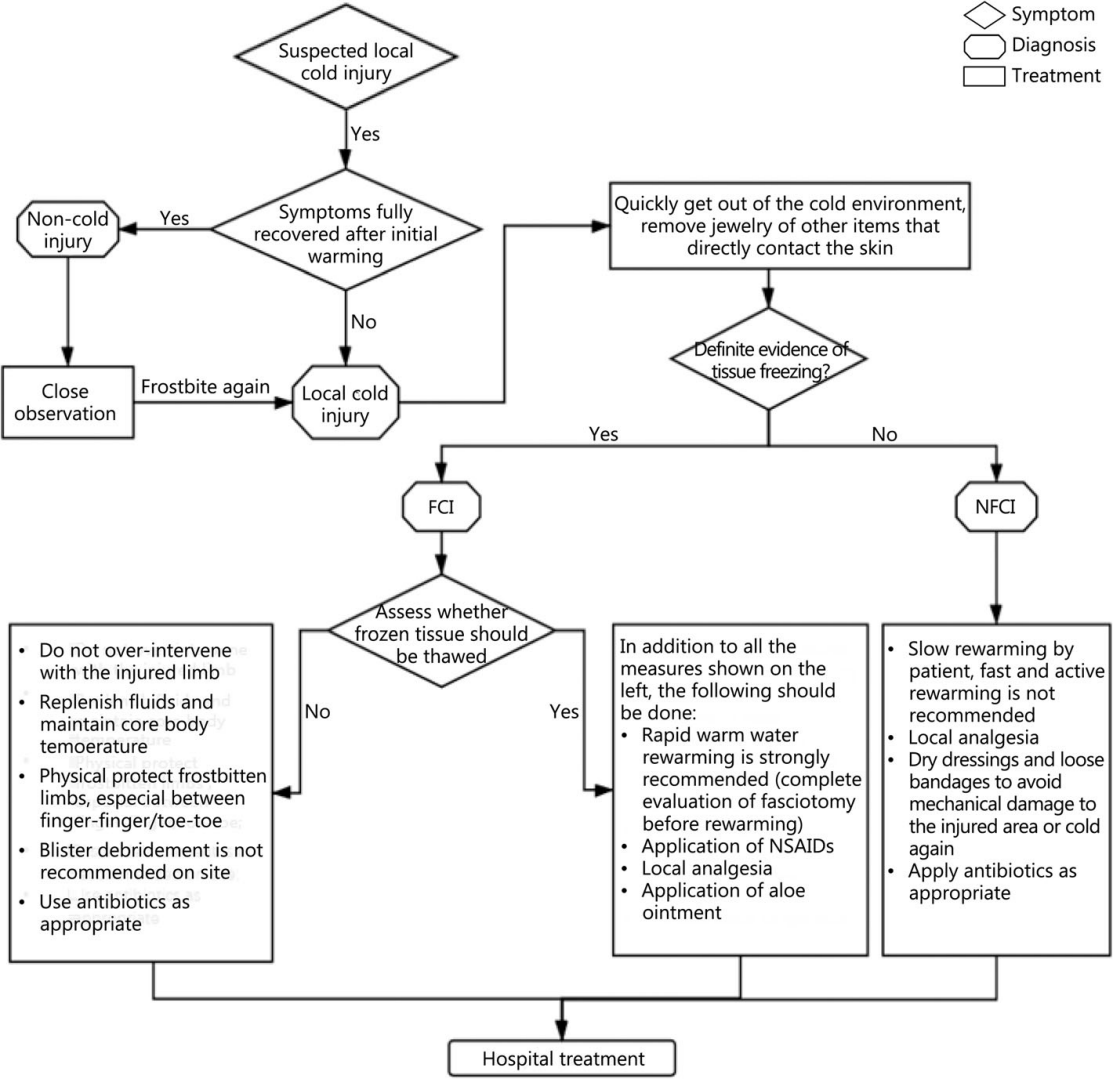
On-site emergency treatments for local cold injury. FCI: Freezing cold injury; NFCI: Non-freezing cold injury

Patients with FCI and NFCI should be removed from the cold environment quickly, and items such as jewelry that are worn close to the body should be removed. After that, the specific type of local cold injury of the patient should be judged quickly (see above), and the corresponding treatment should be given until the patient is sent to the hospital (Fig. 2).

##### NFCI

After getting out of the cold environment, if there is no freezing injury in the affected area, patients can often rewarm slowly on their own. At the same time, to avoid secondary freeze-thaw injury or osteofascial compartment syndrome in patients misdiagnosed as NFCI (who are actually FCI) on-site, rapid and active rewarming (including warm water immersion) is not recommended for NFCI patients [28]. This is also because NFCI is mainly caused by ischemia-reperfusion injury. Rapid rewarming will accelerate this process and lead to a poor prognosis.

In addition, NFCI patients often feel abnormal or no sensation in the affected area. Mechanical injury and cold again should be avoided when these patients are sent to the hospital. Patients with foot injuries should not walk. When there is severe pain in the affected limb, drugs can be given for local analgesia. If there are blisters or ulcers in the affected area, they can be loosely bandaged with dry dressings. Antibiotics can be used prophylactically if there is a risk of infection in the affected area.

##### FCI

It is necessary to decide whether to thaw the frozen tissue first. If thawing could cause the thawed tissue to be frostbitten again in environmental conditions, the damaged tissue should be kept frozen until it can be treated. If natural thawing of damaged tissue occurs, the recurrence of freezing must be avoided.

1. If not appropriate, frozen tissue should not be thawed.

There is no direct evidence that the length of the tissue freezing time is related to the degree of injury [1]. Once it has been determined that the frozen tissue is not suitable for on-site thawing, excessive intervention should not be implemented on the injured limbs. However, it is recommended that adequate fluid be given and the core temperature maintained because hypothermia causes peripheral vasoconstriction, which impairs blood perfusion in the extremities. Currently, there is no study to associate the hydration state of the body with the consequences of frostbite, but it is generally believed that proper infusion of fluids and avoidance of hypoperfusion are important factors promoting recovery from frostbite [5]. If the patient is conscious and has no gastrointestinal reaction, warm fluid should be taken orally.

Physical protection of frostbitten limbs is another important task. The injured parts should be loosely wrapped with dry, nonsticky dressings, and cushions should be applied as protection (especially between fingers and toes). The movement of injured limbs should be reduced as much as possible. In general, blister debridement is not recommended on-site. However, if the blister tension is high and there is a risk of rupture, puncture and aspiration can be implemented on-site, and covering the blister with clean dressings to minimize infection (except bloody blisters). These recommendations are routine, but there is still a lack of research evidence other than a retrospective case analysis [5]. Antibiotics can be used prophylactically if there is a risk of infection.

The above treatment schemes are also applicable to the general treatment of frozen tissue thawing.

(2) Suitability for thawing.

After completely excluding the risk of refreezing, rewarming of frostbitten limbs should be started. It is strongly recommended that a constant temperature water bath be used for rapid rewarming, if available. The water temperature should be maintained between 37 °C and 42 °C (the range of water temperature mentioned in all literature is different) [1, 2, 22]. If there is no thermometer, the uninjured hand of the rescuer can be placed in the water for a period of time (at least 30 s) to ensure that the water will not cause scalding. At the same time, the water may cool quickly after the rewarming process begins, so it should be monitored closely by a thermometer or subjectively assessed by the hands of rescue workers, and the water should be heated to the target temperature continuously and cautiously. The circulation of warm water around the frostbitten tissue will help maintain the right temperature and relieve the pain of the patient. In the process of thawing, the injured limbs can move autonomously, but medical staff should not massage them actively.

Ice and snow should not be rubbed or smeared on the injured part, and the use of other heat sources should be avoided for rewarming (such as baking, using an electric blanket or using other heaters for a long time) because frostbitten tissue is usually numb, so the appropriate temperature is essential to avoid iatrogenic secondary injury.

When the skin of the frostbitten area gradually turns red or purple and becomes soft, it indicates successful rewarming. This process is usually completed within approximately 15 min to 1 h, and it may take more than 1 h for some patients to thaw completely [1, 2, 22]. After that, the damaged tissue should be allowed to dry naturally in warm conditions or absorbed with absorbent paper to minimize further damage.

Before (or during) rewarming, the affected area should be carefully observed, and the risk of osteofascial compartment syndrome should be assessed. The rapid rewarming process of tissue will accelerate ischemia-reperfusion injury and may cause an increase in pressure in the closed soft tissue cavity. It is usually characterized by high muscle tension, obvious pain, limited movement ability and decreased sensation. If there are signs of increased pressure, emergency surgery involving an incision and decompression should be performed to promote limb repair [1].

At the same time, patients may have persistent and severe pain during rewarming, so painkillers (such as NSAIDs or opioid analgesics) should be given to control pain according to the patient’s response and drug supply.

Although there is no direct evidence that any specific NSAIDs (and doses) are significantly beneficial to the prognosis of frostbite, in theory, NSAIDs block the arachidonic acid pathway and reduce the production of prostaglandins and thromboxanes. These mediators can lead to vasoconstriction, skin ischemia and further tissue damage. The commonly used NSAIDs are aspirin (300 mg/d) and ibuprofen (400 mg or 6 mg/kg, 2 times/d) [29]. However, aspirin is generally considered to be an ineffective drug for the treatment of cold injury. As a nonselective inhibitor of prostaglandins, aspirin will inhibit some factors (such as prostacyclin) that are beneficial to wound healing. From this point of view, ibuprofen seems to be the better option. On the other hand, aspirin has anti-platelet agglutination effects. In frostbitten tissue (especially in deep frostbite), vascular thromboembolism caused by vascular endothelial damage is also an important factor in tissue injury. Therefore, aspirin, which has anti-inflammatory, analgesic and anti-platelet aggregation effects, also has merits. At present, there is no specific comparative study between the two. Rescue workers can choose according to the actual situation.

In addition, an observational study with an animal model has shown that aloe ointment can improve the results of frostbite by reducing the formation of prostaglandins and thromboxane [30]. However, topical preparations often do not penetrate deep into tissue. Aloe ointment is theoretically effective only for superficial injury areas. It is worth mentioning that the risk of using aloe preparations is very low. Therefore, when available, aloe ointment can be applied to thawed tissue before it is covered with a dressing.

The recovery of thawed tissue depends to a certain extent on the oxygenation level of the thawed tissue. Although there is a lack of evidence that oxygenation enhances the effectiveness of frostbite treatment, patients with hypoxia (oxygen saturation below normal) or who are above 4000 m in altitude can inhale oxygen through a mask or nasal oxygen tube [2]. Currently, some scholars have proposed that oxygen inhalation should be carried out as soon as possible if there is a cold injury in the body, even at a low altitude (2000–2500 m).

**Recommendation 7: In on-site emergency treatments, in the absence of definite evidence of tissue freezing, all local cold injuries should be treated as NFCIs. (Grade 1C).**

**Recommendation 8: On-site emergency treatment for NFCI should not involve quick and active rewarming. NFCI treatment includes local analgesia, protection of the injured limbs and the use of antibiotics as appropriate. (Grade 1B).**

**Recommendation 9: On-site emergency treatment for FCI should be preceded by a clear decision regarding whether the frozen tissue should be thawed. If the tissue is not suitable for thawing, its treatment should include adequate rehydration, maintenance of core body temperature, protection of the injured limbs, and so on. If tissue thawing can be carried out, in addition to the treatment measures mentioned above, rapid warm water rewarming, local analgesia, and the use of NSAIDs should also be carried out. (Grade 1B).**

### In-hospital treatment

#### Systemic cold injury

For systemic cold injury, the most important and major treatment is still timely and rapid rewarming.

Currently, there are 3 main rewarming methods: in vitro rewarming, minimally invasive rewarming and invasive rewarming. In vitro rewarming includes exercise, drinking sweet and hot drinks, using insulated clothing or quilts and rewarming blankets, raising the ambient temperature, and inhaling hot and humid gas. Minimally invasive rewarming includes bladder lavage and intravenous infusion of warm liquids. Invasive rewarming includes intrathoracic and intraperitoneal perfusion and intravascular rewarming, such as hemofiltration, extracorporeal membrane oxygenation (ECMO) and cardiopulmonary bypass (CPB).

For patients with stable hemodynamics, in vitro and minimally invasive rewarming techniques are recommended. As invasive rewarming may increase the risk of bleeding or thrombosis, it is not recommended. If secondary hypothermia is caused by hypoglycemia, adrenal insufficiency or poisoning, we should actively address the primary disease.

For patients with hemodynamic instability, the airway condition should be thoroughly evaluated and addressed if necessary. If ECMO or CPB is available, one of them can be given, as necessary, at the same time as in vitro rewarming. It has been reported that this can increase the survival rate of patients from less than 37 to 47% - 63% without significant trauma [27]. It is worth noting that the report also mentioned that these patients later had loss of consciousness or neurological dysfunction to some degree. If there is no ECMO or CPB available, thoracic perfusion can be considered. It has also been reported that thoracic perfusion can restore a patient’s autonomic circulatory function within 2 h [26].

Hemofiltration rewarming is recommended for systemic cold injury caused by low-temperature seawater immersion. First, the thermal conductivity of seawater is much greater than that of air, and heat loss in seawater is rapid and extreme. Second, seawater has the properties of high salinity and permeability, which causes not only direct damage to the wound but also electrolyte disorder and dehydration due to high permeability. The main causes are hypothermia, coagulation dysfunction and acidosis, which are collectively called the “death triad”. For treatment of the death triad, hemofiltration has unique advantages: it can increase the temperature of the replacement fluid (up to 42 °C) to offer intravascular core rewarming, and the formula of the replacement fluid can be adjusted to correct electrolyte disorder and acidosis. Blood coagulation function can also be regulated by in vitro anticoagulation technology and calcium ions. At the same time, as a widely developed clinical treatment technology, it has good popularity and accessibility.

In addition, some scholars have pointed out that for patients with severe systemic cold injury (HT IV or core body temperature < 24 °C), the goal of short-term treatment (within 24 h) is to raise the core body temperature to approximately 32 °C and maintain stable vital signs (especially hemodynamics). After that, in addition to related treatments after resuscitation, short-term mild hypothermia therapy (32–34 °C for 24 h) can be considered to reduce brain reperfusion injury and promote the recovery of neurological function [1, 3]. At present, there is no relevant literature involving evidence-based medicine to support this theory.

**Recommendation 10: The key to in-hospital treatment of systemic cold injury still lies in rapid and correct rewarming. For patients with stable hemodynamics, it is recommended that in vitro and minimally invasive rewarming be used; ECMO, CPB and other invasive rewarming methods should be chosen for unstable patients according to the patient’s situation. (Grade 1C).**

#### Local cold injury

Based on the premise that the core body temperature is stable, the treatment of local cold injury should be started immediately. Due to the inconvenience of on-site diagnosis, there may be a misdiagnosis. Therefore, under in-hospital conditions, all patients with local cold injuries should be reevaluated immediately and undergo relevant examinations to make a precise diagnosis and guide the next step of treatment.

##### NFCI

Hospital-specific treatment of NFCI is limited. In addition to general treatment, such as maintaining the stability of water and electrolytes, correcting coagulation disorders, maintaining the stability of hemodynamics and treating complications, selective blister debridement and standardized use of NSAIDs, antibiotics and tetanus preparations should be carried out.

Transparent or turbid liquids in blisters contain prostaglandins and thromboxane, which may damage deep tissue. Blood blisters are thought to be caused by deep tissue damage in the vascular plexus of the skin. The usual practice is to selectively remove transparent blisters while keeping blood blisters intact. However, there is no targeted comparative study to prove whether hemorrhagic blisters should be treated at the same time, and the data in the current literature are not sufficient to make recommendations [17].

Although cold injury is not a traditional infectious injury, there is no literature to specifically support that antibiotics should be used to prevent infection during or after injury. However, some researchers have proposed the use of antibiotics to address edema after thawing. Edema increases the risk of skin infection by gram-positive bacteria. However, there is no research evidence to support this approach. Of course, patients with severe injury, other potential sources of infection, cellulitis or signs and symptoms of sepsis should be given appropriate antibiotic treatment.

The use of tetanus toxoid should be carried out in accordance with standard guidelines [31].

Generally, on the premise of avoiding gastrointestinal mucosal injury,[32] NSAIDs should be used continuously until the wound heals (with doses as mentioned above).

Patients with NFCI usually have refractory pain, which is similar to the neuropathic pain caused by herpes zoster. It is most prominent at night, usually on the soles of the feet, at the base of the toes, or in other affected areas. For the control of such chronic pain, a British military study recommended the use of amitriptyline (or other tricyclic antidepressants). These drugs can relieve chronic pain by acting on central opioid receptors. Generally, amitriptyline can produce obvious curative effects after 7–10 days of treatment, which may be due to the inhibitory effect of amitriptyline on anxiety caused by long-term pain [6]. For healthy adults, the initial dose is recommended to be 50 mg/day, and if necessary, it can be gradually increased to a maximum of 150 mg/day. In addition, this study does not recommend any form of sympathetic block, which usually leads to the deterioration of symptoms in the middle and later stages [6].

##### FCI

The above general treatment for NFCI (including blister debridement, NSAIDs, antibiotics, tetanus) is also applicable to FCI. In addition, due to the particularity of FCI, its in-hospital treatment is often more complicated.

For moderate to severe (deep tissue injury) FCI, one of the leading causes is local ischemia caused by thromboembolism after endothelial cell damage. Therefore, intravenous or intra-arterial infusion of tissue plasminogen activator (tPA) within 24 h after thawing may save some or all at-risk tissues. A single-center retrospective study showed that the amputation rate of patients who did not receive tPA treatment was 41%, while that of patients who received tPA treatment within 24 h was only 10% [32].

Thrombolysis should be performed in a hospital with intensive care capabilities. At present, the main treatment for preventing local thrombosis is heparin combined with thrombolysis, in which heparin is recommended as an adjunct to reduce the recurrence of microvascular thrombosis [33].

However, not all patients are suitable for thrombolytic therapy. When the patient is complicated with trauma or intracranial hemorrhage or when the patient has been thawed for more than 24 h, the risk of thrombolytic therapy often outweighs its benefit. For such patients, the selective use of vasodilators may be more conducive to recovery from the disease.

Currently, prostaglandin Emael-1 (PGE-1), prostaglandin analogue iloprost, nitroglycerin, nifedipine and buflomedil have been used as adjuvant drugs in the treatment of FCI [34].

In Europe, an increasing amount of data support the use of iloprost. Some studies showed that the rate of finger and toe amputations decreased significantly [34]: after rapid rewarming and administration of 250 mg aspirin and 400 mg buflomedil, 47 patients with severe frostbite (at high risk of finger/toe amputation) were randomly assigned to a buflomedil group (group A), an iloprost group (group B), and an iloprost plus tPA group (group C). All patients received treatment for 8 days. The total amputation rate of the iloprost group (group B) was 0%, which was the lowest, compared with 16% for the tPA group (group C) and 60% for the buflomedil group (group A). However, as the degree of frostbite in the tPA group (group C) was more severe than that in the other two groups, the beneficial effect of tPA was not ruled out.

As mentioned above, there are limited data to show the effectiveness of the treatment. Iloprost is the only vasodilator with sufficient evidence to support its use. Therefore, if proper monitoring facilities are available, iloprost should be regarded as a first-line drug for patients with deep frostbite.

**Recommendation 11: In-hospital treatment of NFCI generally includes selective blister debridement and standardized use of NSAIDs, antibiotics and tetanus. (Grade 1B).**

**Recommendation 12: In-hospital treatment of FCI is generally the same as that of NFCI. In addition, thrombolysis or vasodilators should be chosen according to the patient’s condition. Heparin can be used as an adjuvant. (Grade 1B).**

### Special treatment and psychological intervention

#### Hyperbaric oxygen therapy

As hyperbaric oxygen therapy increases tissue oxygenation, many types of wound studies suggest that wound healing is accelerated or more complete following hyperbaric oxygen therapy [35]. On the other hand, because hyperbaric oxygen increases the oxygen tension in the blood, the treatment is usually effective only when the blood supply of the distal tissue is adequate. There is no discernible effect in patients with severe frostbite.

In animal experiments related to cold injury, hyperbaric oxygen therapy is not ideal and often contradictory. In one study, the hind legs of 64 rabbits were treated with hyperbaric oxygen therapy after frostbite, and the affected area was allowed to rewarm slowly. The study showed no significant effect on tissue survival. After that, the same researcher repeated the experiment, and the affected area was quickly rewarmed; however, the results remained unchanged [1]. Another study showed that the tissue survival rate of rabbits was improved if hyperbaric oxygen was used for 2 h a day, and if the therapy was given within 24 h after cold injury, the tissue survival rate was higher [10]. However, due to the different limiting conditions of the two experiments and the lack of damage uniformity, it is difficult to make an accurate judgment of their effects.

Currently, the overall data are not sufficient to support hyperbaric oxygen as a routine method for the treatment of frostbite.

#### Sympathectomy

Because blood flow partly depends on sympathetic tension, some scholars have proposed that chemical or surgical sympathectomy should be used in the early stage of cold injury (especially deep frostbite) to accelerate the formation of the tissue necrosis boundary and retain the function of the remaining tissue to the greatest extent. In the rat lower limb model, early removal of the sympathetic nerve (within 24 h of cold injury) can reduce tissue defects. However, the effect is not good after 24 h [33]. In addition, there are delayed symptoms in patients with frostbite, such as pain, abnormal sensation and redness of the skin. The results of chemical or surgical sympathectomy for these symptoms vary. At the same time, there are not enough clinical data to prove that it can ultimately reduce tissue defects [17].

#### Surgical amputation

After frostbite, it may take 1–3 months for the complete boundary of tissue necrosis to form. However, angiography, Technetium-99 bone scans or MRI can be used to help determine the scope of surgery at an early stage [36]. A retrospective study by Cauchy et al. [37] showed that Technetium-99 scans could even accurately predict the level of amputation in 84% of cases within 2 days after injury.

During this period, if the patient shows symptoms and signs of sepsis caused by frostbite tissue infection and the treatment team determines that stable vital signs could not be maintained through active medical treatment, amputation should be chosen quickly [38]. Because unnecessary surgery or premature surgical intervention may lead to serious adverse consequences, the timing and necessity of an operation should be fully evaluated before amputation at any site.

#### Cold sensitivity therapy

For patients with cold injury, the increased cold sensitivity caused by the disease often lasts for months or even a lifetime. Moderate and severe NFCI patients are more common. Even in patients with mild or subclinical cold injury, increased cold sensitivity is often reported. This symptom can be so severe that it affects daily life. Some vasodilators, such as nifedipine, may have some effect on this kind of patient, but there are not enough clinical data to support it. In addition, some researchers have pointed out that this sensitivity can be gradually reduced or even disappear by changing the living environment (such as by working indoors in heated buildings) to minimize cold exposure. Therefore, living and working in tropical or subtropical climates may be the best choice, and it has been reported that some patients have achieved complete clinical recovery after 2–3 years [2].

#### Treatment with traditional Chinese medicine

Some traditional Chinese medicines have specific effects on mild to moderate cold injury. In one study, a mouse foot frostbite model was made by continuous freezing with ethanol, and the injury was treated with *Taxillus chinensis* for 6 days (without rewarming after injury). The degree of tissue damage was significantly lower than that of the control group (which received only rapid rewarming in a water bath after injury). The mechanism may be related to the improvement of microcirculation and the inhibition of inflammatory factors by choline and acetylcholine amines in *Taxillus chinensis* [39].

In addition to *Taxillus chinensis*, there are also reports on the treatment of cold injury with Yunnan Baiyao and other traditional Chinese medicines [40]. However, overall, there are few studies on the treatment of cold injury with traditional Chinese medicine. Therefore, no specific suggestions will be made here.

#### Psychological intervention therapy

Patients with cold injury (especially severe cold injury) suffer a severe mental blow, complicated by prolongation of the course of the disease. Especially if amputation is needed after frostbite, there will be permanent defects in body appearance and corresponding function loss. This not only affects normal labor, life and social interactions but also causes severe and lasting psychological trauma, and the potentially bad mental state may affect a person’s life. Therefore, psychological intervention for patients with cold injury can help to improve the therapeutic effect of treatments for frostbite or basic diseases, effectively enhance patients’ confidence in overcoming the disease, enhance their sense of security, and eliminate their fear.

**Recommendation 13: Hyperbaric oxygen therapy, sympathectomy and traditional Chinese medicine treatment lack sufficient clinical data support and should not be used as routine treatments for cold injury. (Grade 2B).**

**Recommendation 14: The medical team should strictly evaluate the patient’s condition and perform surgical amputation if necessary. (Grade 1B).**

**Recommendation 15: Necessary psychological intervention should be implemented in patients with cold injury (especially moderate and severe cold injury). (Grade 1C).**

In general, the focus of diagnosis and treatment of cold injury is early diagnosis, precise classification, timely selection of corresponding rewarming methods and treatment measures after getting out of the cold environment and immediate transfer to the hospital. In most cases, improving awareness about cold injury prevention and taking relevant active measures for prevention can prevent its occurrence to the greatest extent. This is the best solution.

## Supplementary Information

**Additional file 1.** Staging of local freezing cold injury (refers to burn grade).

**Additional file 2.** Traditional classification of systemic cold injury.

**Additional file 3.** Statistics of patients hospitalized with cold injury in Finland.

## Data Availability

Not applicable.

## Abbreviations

ACCP: American College of Chest Physicians;
CPB: Cardiopulmonary bypass;
CPR: Cardiopulmonary resuscitation;
ECMO: Extracorporeal membrane oxygenation;
FCI: Freezing cold injury;
NFCI: Non-freezing cold injury;
WMS: Wilderness Medical Society

## References

[1] Heil K, Thomas R, Robertson G, Porter A, Milner R, Wood A (2016). Freezing and non-freezing cold weather injuries: a systematic review. Br Med Bull.

[2] Imray CH, Oakley EH (2005). Cold still kills: cold-related illnesses in military practice freezing and non-freezing cold injury. J R Army Med Corps.

[3] Wang HS, Han JS (2014). International research status of war trauma rescue and treatment in cold region. Med J Chin PLA.

[4] Cauchy E, Chetaille E, Marchand V, Marsigny B (2001). Retrospective study of 70 cases of severe frostbite lesions: a proposed new classification scheme. Wilderness Environ Med..

[5] McIntosh SE, Hamonko M, Freer L, Grissom CK, Auerbach PS, Rodway GW (2011). Wilderness medical society practice guidelines for the prevention and treatment of frostbite. Wilderness Environ Med.

[6] Imray CH, Richards P, Greeves J, Castellani JW (2011). Nonfreezing cold-induced injuries. J R Army Med Corps.

[7] Xue CJ, Xia YJ, Liu JY. Clinical research progress on cold injury. China Occup Med. 2015; 42(3):338–340, 44. [Article in Chinese]..

[8] Durrer B, Brugger H (2003). Syme D; international commission for mountain emergency medicine. The medical on-site treatment of hypothermia: ICAR-MEDCOM recommendation. High Alt Med Biol.

[9] O'Donnell FL, Taubman SB (2016). Update: cold weather injuries, active and reserve components, U. S. armed forces, July 2011-June 2016. MSMR..

[10] Daanen HA, van der Struijs NR (2005). Resistance index of frostbite as a predictor of cold injury in Arctic operations. Aviat Space Environ Med.

[11] Ervasti O, Juopperi K, Kettunen P, Remes J, Rintamäki H, Latvala J (2004). The occurrence of frostbite and its risk factors in young men. Int J Circumpolar Health..

[12] Liu YE, Liu XW, Yin XH (2015). Cold damage among soldiers in cold area of Northeast China:a cross-sectional survey. Chin J Public Health.

[13] Harirchi I, Arvin A, Vash JH, Zafarmand V (2005). Frostbite: incidence and predisposing factors in mountaineers commentary. Br J Sports Med.

[14] Tian P, Li C, Wang H, Wang CQ, Du WL. Analysis of 59 cases of frostbite in plain region. Chin J Burn. 2009; 25(5):377–379. [Article in Chinese].. 19951563

[15] Hall A, Evans K, Pribyl S (2010). Cold injury in the United States military population: current trends and comparison with past conflicts. J Surg Educ.

[16] Sokolov V, Biryukov A, Chmyrev I, Tarasenko M, Kabanov P (2017). Burns and frostbite in the red Army during world war II. Mil Med Res.

[17] Murphy JV, Banwell PE, Roberts AH, McGrouther DA (2000). Frostbite: pathogenesis and treatment. J Trauma.

[18] Juopperi K, Hassi J, Ervasti O, Drebs A, Näyhä S (2002). Incidence of frostbite and ambient temperature in Finland, 1986-1995. A national study based on hospital admissions. Int J Circumpolar Health..

[19] Niu YM, Xia YJ. Retrospective study on 397 cases of frostbite. Chin J Indust Med. 2013; 26(5):338–340. [Article in Chinese]..

[20] DeGroot DW, Castellani JW, Williams JO, Amoroso PJ (2003). Epidemiology of U. S. Army cold weather injuries, 1980-1999. Aviat Space Environ Med.

[21] Dobnikar U, Kounalakis SN, Mekjavic IB (2009). The effect of exercise-induced elevation in core temperature on cold-induced vasodilatation response to toes. Eur J Appl Physiol.

[22] McIntosh SE, Opacic M, Freer L, Grissom CK, Auerbach PS, Rodway GW (2014). Wilderness medical society practice guidelines for the prevention and treatment of frostbite: 2014 update. Wilderness Environ Med..

[23] Leominster E (2000). Emollients in the prevention of frostbite. Int J Circumpolar Health.

[24] Carlsson D, Pettersson H, Burström L, Nilsson T, Wahlström J (2016). Neurosensory and vascular function after 14 months of military training comprising cold winter conditions. Scand J Work Environ Health.

[25] Ministry of Defence(UK). Heat illness and cold injury: prevention and management, part 2: guidance. Joint services publication 539.2019; (ver.3.1):4-6.

[26] Brown DJ, Brugger H, Boyd J, Paal P (2012). Accidental hypothermia. New England J Med.

[27] Paul P, Brugger H, Boyd J (2012). Accidental hypothermia. N Engl J Med.

[28] Glennie JS, Milner R (2014). Non-freezing cold injury. J R Nav Med Serv.

[29] Rainsford KD (2009). Ibuprofen: pharmacology, efficacy and safety. Inflammopharmacology..

[30] Grieve AW, Davis P, Dhillon S, Richards P, Hillebrandt D, Imray CH (2011). A clinical review of the management of frostbite. J R Army Med Corps.

[31] Emergency Physician Branch Of Chinese Medical Doctor Association, Emergency Medicine Committee of the people's Liberation Army of China, Beijing Society for Emergency Medicine, et al. Expert consensus on prophylaxis, diagnosis and management of tetanus among adults. Chin J Emerg Med. 2018; 27(12):1323–1332. [Article in Chinese]..

[32] Bruen KJ, Ballard JR, Morris SE, Cochran A, Edelman LS, Saffle JR (2007). Reduction of the incidence of amputation in frostbite injury with thrombolytic therapy. Arch Surg.

[33] Saemi AM, Johnson JM, Morris CS (2009). Treatment of bilateral hand frostbite using transcatheter arterial thrombolysis after papaverine infusion. Cardiovasc Intervent Radiol.

[34] Bruen KJ, Gowski WF (2009). Treatment of digital frostbite: current concepts. J Hand Surg Am.

[35] Jurkovich GJ. Environmental cold-induced injury. Surg Clin North Am. 2007; 87(1):247–267. viii. 10.1016/j.suc.2006.10.003 17127131

[36] Cauchy E, Cheguillaume B, Chetaille E (2011). A controlled trial of a prostacyclin and rt-PA in the treatment of severe frostbite. N Engl J Med.

[37] Cauchy E, Marsigny B, Allamel G, Verhellen R, Chetaille E (2000). The value of technetium 99 scintigraphy in the prognosis of amputation in severe frostbite injuries of the extremities: a retrospective study of 92 severe frostbite injuries. J Hand Surg Am..

[38] Purkayastha SS, Bhaumik G, Chauhan SK, Banerjee PK, Selvamurthy W (2002). Immediate treatment of frostbite using rapid rewarming in tea decoction followed by combined therapy of pentoxifylline, aspirin & vitamin C. Indian J Med Res.

[39] Wang XY, Tan L, Huang Z. Study of traditional Chinese Medicine Taxillusi chinensis (DC.) in the treatment of cold injury. Chin Tradit Patent Med. 2011; 33(11):1990–1993. [Article in Chinese].

[40] Xiamixikamaer, Gulibositan. The clinical application of Yunnan Baiyao. Herald of Medicine. 2003; (S1):282-283. [Article in Chinese].

