# Supplementary material for: Expert consensus on the prevention, diagnosis and treatment of cold injury in China, 2020
Source: Mil Med Res. 2021 Jan 21;8:6. doi: 10.1186/s40779-020-00295-z (PMC7818913; doi:10.1186/s40779-020-00295-z)
Supplement: Supplementary file 1 — Additional file 1. Staging of local freezing cold injury (refers to burn grade). [file 40779_2020_295_MOESM1_ESM.docx]

**Additional file 1.** Staging of local freezing cold injury (refers to burn grade)

| Stage | Content |
| --- | --- |
| Level 1 | Often accompanied by skin numbness and erythema. White or yellow, hard and slightly raised patches may appear in the damaged area. There is no gross tissue necrosis, and there might be slight epidermis exfoliation. Mild edema common. |
| Level 2 | Blisters are formed on the surface of the skin. There is clear or milky white liquid in the blisters. There are erythema and edema around the blisters. The lesions reach the dermis. |
| Level 3 | The whole layer of the skin and subcutaneous tissue are involved. The main feature is the necrosis of the whole layer of the skin. The skin is cyanotic or purplish red, then changes to black, and sensation disappears. There may be hemorrhagic blisters. |
| Level 4 | It has completely penetrated the dermis, involving the subcutaneous tissue with relatively few blood vessels, and necrosis extends to the level of muscle and bone. Complete loss of sensation and motor function. |
