# Supplementary material for: Expert consensus on the prevention, diagnosis and treatment of cold injury in China, 2020
Source: Mil Med Res. 2021 Jan 21;8:6. doi: 10.1186/s40779-020-00295-z (PMC7818913; doi:10.1186/s40779-020-00295-z)
Supplement: Supplementary file 2 — Additional file 2. Traditional classification of systemic cold injury. [file 40779_2020_295_MOESM2_ESM.docx]

**Additional file 2.** Traditional classification of systemic cold injury

| Grade | Corresponding core body temperature |
| --- | --- |
| Mild | 32℃<T<35℃ |
| Moderate | 28℃<T<32℃ |
| Severe | 20℃<T<28℃ |
| Profound | T<20℃ |
