# Supplementary material for: Expert consensus on the prevention, diagnosis and treatment of cold injury in China, 2020
Source: Mil Med Res. 2021 Jan 21;8:6. doi: 10.1186/s40779-020-00295-z (PMC7818913; doi:10.1186/s40779-020-00295-z)
Supplement: Supplementary file 3 — Additional file 3. Statistics of patients hospitalized with cold injury in Finland. [file 40779_2020_295_MOESM3_ESM.docx]

**Additional file 3.** Statistics of patients hospitalized with cold injury in Finland

| Age(yr) | Males | Females | Both sexes | |
| --- | --- | --- | --- | --- |
|  | N | N | N | % |
| 0-9 | 8 | 3 | 11 | 0.9 |
| 10-19 | 87 | 19 | 106 | 8.3 |
| 20-29 | 154 | 10 | 161 | 12.9 |
| 30-39 | 134 | 21 | 155 | 12.2 |
| 40-49 | 205 | 31 | 263 | 18.5 |
| 50-59 | 191 | 16 | 207 | 16.2 |
| 60-69 | 162 | 18 | 180 | 14.1 |
| 70-79 | 110 | 31 | 141 | 11.1 |
| 80+ | 44 | 31 | 75 | 5.9 |
| Total | 1095 | 180 | 1275 | 100.0 |
